# Dr Faustine Engelbert Ndugulile (1969–2024): A visionary leader and champion for health equity

**DOI:** 10.4102/jphia.v16i1.1316

**Published:** 2025-03-27

**Authors:** Aisa Muya, Donatus Mutasingwa, Edmund Rutta, Ellen Mkondya-Senkoro, Eliud Wandwalo, Mohamed A Mohamed, Mpoki Ulisubisya, Mwidimi Ndosi

**Affiliations:** 1 Amref Health Africa Tanzania, Dar es Salaam, Tanzania; 2 Department of Family Medicine, Oak Valley Health, Toronto, Canada; 3 Department of Family and Community Medicine, University of Toronto, Toronto, Canada; 4 United States Agency for International Development, Washington, DC, United States of America; 5 Benjamin W. Mkapa Foundation, Dodoma, Tanzania; 6 The Global Fund to Fight HIV, Tuberculosis and Malaria, Geneva, Switzerland; 7 East, Central and Southern Africa Health Community (ECSAHC), Arusha, Tanzania; 8 Muhimbili Orthopaedic and Neurosurgery Institute (MOI), Dar es Salaam, Tanzania; 9 School of Health and Social Wellbeing, University of the West of England, Bristol, United Kingdom

The global health community mourns the loss of Dr Faustine Engelbert Ndugulile, a Tanzanian public health luminary and visionary leader. He was renowned for his work in strengthening health systems and advancing global health equity. His untimely passing leaves an indelible void in medicine, public health and governance.

Dr Ndugulile’s career epitomised the intersection of evidence-based practice, public service and transformative leadership. His recent election as WHO Regional Director for Africa, a role he was due to assume in February 2025, marked a crowning achievement – one that reflected his lifelong dedication to health equity and innovation.

An African proverb states ‘Uwile umth’omkhulu’ – ‘the great tree has fallen’. Although not an elder in years, Dr Ndugulile was a pillar of knowledge, integrity and unity. His unwavering commitment to improving health outcomes for marginalised populations inspired all who knew him.

Dr Ndugulile earned his medical degree in 1997 from the University of Dar es Salaam’s School of Medicine (now Muhimbili University of Health and Allied Sciences), where he served as Secretary General of the Tanzanian Medical Students Association (TAMSA). He later obtained a Master of Medicine in Microbiology and Immunology (2001), a Master of Public Health (2010) and a Bachelor of Laws (2022), underscoring his belief in multidisciplinary solutions to health and human rights challenges.

His career spanned clinical medicine, global health and policy. As Resident Advisor for the United States Centers for Disease Control and Prevention (US CDC) in South Africa (2007–2010), he strengthened disease surveillance and laboratory systems in multiple African countries. He played a key role in the Global and African TB Caucuses, shaping policies for tuberculosis prevention and treatment.

Elected as the Member of Parliament for Kigamboni in 2010, he championed health legislation, maternal and child health and social service reforms. As Tanzania’s Deputy Minister for Health (2017–2020), he emphasised science-driven interventions in the country’s coronavirus disease 2019 (COVID-19) response. Later, as Minister of Communication and Information Technology (2021), he spearheaded the Digital Tanzania Project, emphasising digital innovation for development.

His ability to bridge medicine, governance and technology positioned him as a unique and impactful leader on the global stage. He built partnerships across sectors and mentored young leaders – guided by the African proverb: ‘If you want to go fast, go alone. If you want to go far, go together’.

We extend our deepest condolences to his wife, Dr Magdalena Lyimo; his children, Martha and Melvin; and his extended family, friends and colleagues. Dr Faustine Ndugulile will be remembered as a visionary leader, a compassionate physician and a steadfast advocate for global health equity.

The authors would like to acknowledge the contribution of the Muhimbili University College of Health Sciences, Alumni Class of 1995 and 1996.

